# Shiga Toxin 1, as DNA Repair Inhibitor, Synergistically Potentiates the Activity of the Anticancer Drug, Mafosfamide, on Raji Cells

**DOI:** 10.3390/toxins5020431

**Published:** 2013-02-21

**Authors:** Maurizio Brigotti, Valentina Arfilli, Domenica Carnicelli, Laura Rocchi, Cinzia Calcabrini, Francesca Ricci, Pasqualepaolo Pagliaro, Pier Luigi Tazzari, Roberta R. Alfieri, Pier Giorgio Petronini, Piero Sestili

**Affiliations:** 1 Department of Experimental, Diagnostic and Specialty Medicine, University of Bologna, Via San Giacomo 14, Bologna 40126, Italy; E-Mails: arfilli.v@libero.it (V.A.); domenica.carnicelli@unibo.it (D.C.); laura.rocchi6@unibo.it (L.R.); 2 Department of Biomolecular Sciences, University of Urbino “Carlo Bo”,Via Saffi 2, Urbino 61029, Italy; E-Mails: cinzia.calcabrini@uniurb.it (C.C.); piero.sestili@uniurb.it (P.S.); 3 Immunohematology and Transfusion Center, S. Orsola-Malpighi Hospital, Via Massarenti 9, Bologna 40138, Italy; E-Mails: francesca.ricci@aosp.bo.it (F.R.); pasqualepaolo.pagliaro@aosp.bo.it (P.P.); immunologia@aosp.bo.it (P.L.T.); 4 Department of Clinical and Experimental Medicine, University of Parma, Via Volturno 39, Parma 43126, Italy; E-mails: roberta.alfieri@unipr.it (R.R.A.); piergiorgio.petronini@unipr.it (P.G.P.)

**Keywords:** Shiga toxin 1, Gb3Cer/CD77-expressing lymphomas, mafosfamide, DNA repair, autologous bone marrow transplantation

## Abstract

Shiga toxin 1 (Stx1), produced by pathogenic *Escherichia coli*, targets a restricted subset of human cells, which possess the receptor globotriaosylceramide (Gb3Cer/CD77), causing hemolytic uremic syndrome. In spite of the high toxicity, Stx1 has been proposed in the treatment of Gb3Cer/CD77-expressing lymphoma. Here, we demonstrate in a Burkitt lymphoma cell model expressing this receptor, namely Raji cells, that Stx1, at quasi-non-toxic concentrations (0.05–0.1 pM), inhibits the repair of mafosfamide-induced DNA alkylating lesions, synergistically potentiating the cytotoxic activity of the anticancer drug. Conversely, human promyelocytic leukemia cells HL-60, which do not express Gb3Cer/CD77, were spared by the toxin as previously demonstrated for CD34+ human progenitor cells, and hence, in this cancer model, no additive nor synergistic effects were observed with the combined Stx1/mafosfamide treatment. Our findings suggest that Stx1 could be used to improve the mafosfamide-mediated purging of Gb3Cer/CD77+ tumor cells before autologous bone marrow transplantation.

## 1. Introduction

Shiga toxin 1 (Stx1), produced by some pathogenic *Escherichia coli* strains [1], damages cellular nucleic acids by removing a specific adenine from 28S rRNA in ribosomes [2] and multiple adenines from DNA *in vitro* [3]. This leads to inhibition of protein synthesis in the cytoplasm and formation of apurinic sites in the nucleus of target eukaryotic cells [4,5]. At quasi-non-toxic concentrations, Stx1 also blocks the DNA repair of lesions induced by oxidizing and alkylating agents [6]. This effect is unrelated either to inhibition of protein synthesis or to depletion of cellular antioxidant defenses and is likely to derive from direct interactions with cellular DNA repair machinery [6]. It should be noted that, although in these conditions Stx1 reaches the nucleoplasm and the cytoplasm, DNA was undamaged and translation little affected [6]. Thus, the spectrum of toxin actions in sensitive cells depends on the concentration of the toxin, being considered a DNA repair inhibitor at low dosage. 

Stx1 targets normal [1,7] and cancer [8,9] human cells expressing globotriaosylceramide (Gb3Cer/CD77) on their membrane. In hematological cells, Gb3Cer/CD77 is expressed on the surface of a narrow range of committed B lymphocytes present in germinal centers, as well as on the associated B-cell lymphomas, such as Burkitt lymphoma [10]. In particular, Gb3Cer/CD77 was found to be highly accumulated in lymphoma cell lines [11]. This receptor has also been detected in biopsies from 70% of patients with follicular lymphoma and 30%–40% of patients with small lymphocytic lymphoma [10,11]. 

About 50% of patients with low-grade or intermediate-grade non-Hodgkin lymphoma are treated with high-dose chemotherapy followed by autologous bone marrow transplantation. In this context, a widely used drug is mafosfamide, an alkylating agent forming DNA cross-links and DNA strand breaks, inhibiting DNA synthesis and triggering apoptosis in target cells [12]. Mafosfamide is a stable salt of 4-OH-cyclophosphamide that does not require metabolic activation. This renders the drug suitable for the elimination of cancer cells before autologous bone marrow transplantation [12]. Stx1 has also been proposed as a selective purging agent against Gb3Cer/CD77+ cells in this context, as this toxin has shown no toxicity against CD34+ human progenitor cells, which do not express Gb3Cer/CD77 [10]. However, the safety of Stx1 as a purging agent in an *ex vivo* setting has been questioned, since Gb3Cer/CD77 is also expressed by cerebral, intestinal and renal endothelia and by renal cells in humans [1]. The consequence of the damaging effects of Stx1 on these cells is the development of hemorrhagic colitis and of the life-threatening sequela hemolytic uremic syndrome, the main cause of acute renal failure in early childhood [13,14]. Although residual toxin in the purged marrow could be removed by extensive washing or by neutralizing antibodies [10], the risk of toxicity is still fairly high since Stx1 acts on these cells at picomolar concentrations. We investigated here the effects of lower concentrations of Stx1 on a Burkitt lymphoma cell line, namely Raji, which expresses Gb3Cer/CD77 [15], and on the human myeloid leukemia cells HL-60, which do not harbor even trace amounts of the receptor on their membrane [16]. DNA repair of lesions induced by the alkylating agent mafosfamide in Raji cells was inhibited by Stx1, resulting in synergistic cooperation between the bacterial toxin and the purging agent in the elimination of Raji cancer cells. Conversely, DNA repair and protein synthesis were unaffected in HL-60 cells treated with Stx1, which did not elicit any toxic effect on these cells either alone or in combination with mafosfamide.

## 2. Results

**Figure 1:**
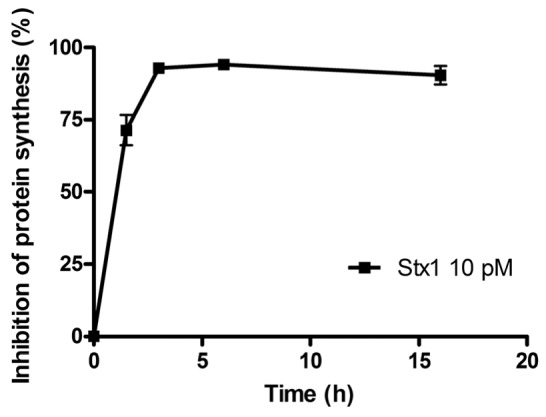
Time course of inhibition of protein synthesis in Raji cells treated with 10 pM Shiga toxin 1 (Stx1). The SD values (*n* = 3) of single points are indicated.

The time course of the inhibition of protein synthesis in Raji cells incubated with 10 pM Stx1 is shown in Figure 1. Raji cells were very sensitive to the damaging effects induced by Stx1, which was internalized within 90 min, as indicated by the almost total inhibition of translation caused by the toxin-induced ribosomal lesions.

**Figure 2:**
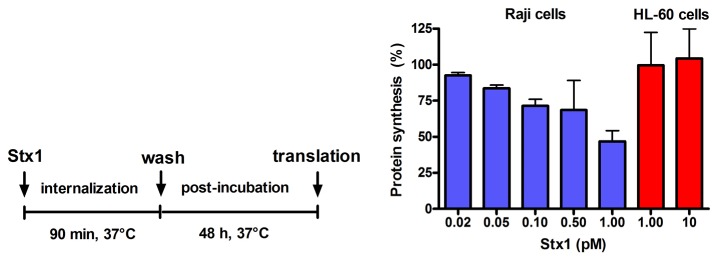
Effect of Stx1 on protein synthesis in Raji cells or HL-60 cells. Cells (1 × 10^6^) were either untreated or treated with Stx1, as described in the Figure. Post-incubation was performed after a single wash followed by resuspension in 1.5 mL complete medium. At the end of post-incubation, the rate of protein synthesis was measured, as described in Experimental section. The [^3^H]leucine incorporated by untreated Raji cells or untreated HL-60 cells was 16,358 ± 3503 dpm (*n* = 3) or 20703 ± 2691 dpm (*n* = 3), respectively. Data are expressed as percent of translation with respect to controls; the SD values (*n* = 3) of single points are indicated.

**Figure 3:**
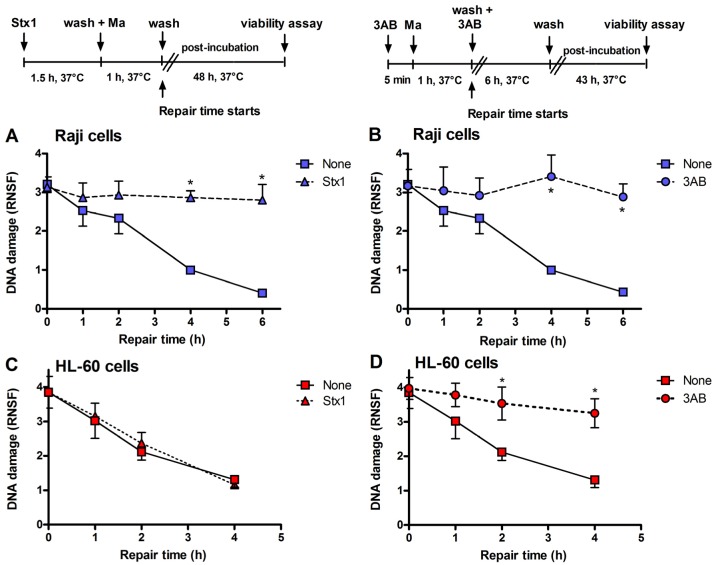
Inhibition of DNA repair induced by Stx1 and 3-aminobenzamide (3AB) of mafosfamide-dependent alkylative lesions. Raji cells were either untreated or treated with 0.1 pM Stx1 (**A**) or 5 mM 3AB (**B**), as described in the upper panels. HL-60 cells were either untreated or treated with 0.1 pM Stx1 (**C**) or 5 mM 3AB (**D**), as described in the upper panels. After 5 min centrifugation at 200 × *g* and washing, pelleted cells resuspended in 1.5 mL complete medium were either untreated or treated with 5 μg/mL mafosfamide (Ma) and washed. Then, cells were seeded in 1.5 mL complete medium in 3.5 cm diameter dishes for 48 h at 37 °C. In the case of the diffusible 3AB, washings were performed in the presence of the DNA repair inhibitor. After mafosfamide treatment, cells were allowed to repair nuclear DNA lesions in drug-free culture medium. The level of residual DNA strand breakage was determined with FHA at increasing time intervals (up to 6 h). Data are expressed as relative nuclear spreading factor (RNSF) (see Experimental section) and are the mean ± SEM from at least four separate experiments. * *p* < 0.001 as compared to cells exposed to mafosfamide alone and allowed to repair for the indicated times (*t*-test).

Incubation of Raji cells for 90 min at 37 °C with lower concentrations of Stx1 (0.02–1 pM) followed by 48 h post-incubation in toxin-free medium caused a dose-dependent inhibition of translation (Figure 2). Under the same conditions, Stx1, tested at 10-fold higher concentration, did not elicit any effect on translation in HL-60 cells (Figure 2). In Raji cells, lowering the toxin concentration at 0.1 pM, induced poor toxic effects, as protein synthesis was about 25% impaired (Figure 2) and no damaging effects on DNA were detected by the fast halo assay (FHA) [17] (not shown). This sensitive assay has been employed to demonstrate for the first time the damaging effects of Shiga toxins on nuclear DNA in human endothelial cells treated with higher toxin concentrations [4,5]. When Raji cells or HL-60 cells were challenged with the alkylating drug mafosfamide (5 μg/mL) in the presence of Stx1 at the same low-toxic 0.1 pM concentration (experimental setting in Figure 3), the repair of mafosfamide-induced DNA lesions, assessed by FHA, was completely inhibited in Raji cells (Figure 3A) and fully efficient in HL-60 cells (Figure 3C).

A similar DNA repair impairment was observed in Raji cells and HL-60 cells by substituting 3-aminobenzamide (3AB, 5 mM) for Stx1 (Figure 3B,D). 3AB is a well known inhibitor of the enzyme poly(ADP-ribose)polymerase (PARP), which is involved in DNA repair of alkylative lesions. In this case, the experimental setting was modified to allow the presence of the diffusible inhibitor during the repair time (see right panel, Figure 3). The extents of DNA repair inhibition over time obtained in Raji cells with Stx1 and 3AB were comparable (Figure 3). 

**Figure 4:**
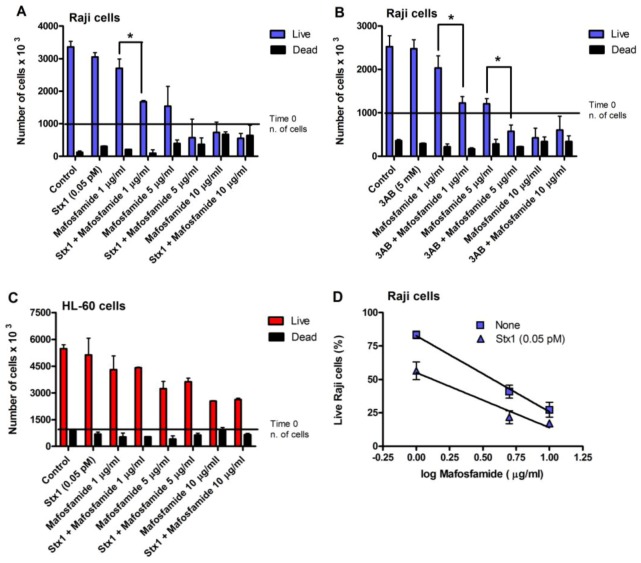
Raji cell viability after treatment with mafosfamide in combination with Stx1 (0.05 pM) or 3AB (5 mM). Representative experiments performed with Raji cells (**A**, **B**) or with HL-60 cells (**C**) as negative control. Cells were treated, as described in the panels of Figure 3. At the end of post-incubation, cell survival was assessed with a trypan blue permeation assay, as described in Experimental section. The SD values (*n* = 3) of single points are indicated. * *p* < 0.05 as compared to cells exposed to mafosfamide alone (*t*-test); (**D**) The curves were obtained by plotting the mean percent of live Raji cells obtained from three independent experiments with Stx1 against the logarithm of mafosfamide concentrations. The SD values of single points are indicated.

With the same experimental settings (Figure 3), we also evaluated Raji cell viability, measured with the trypan blue dye exclusion test, after treatment with different concentrations of mafosfamide (1–10 μg/mL) in the absence and in the presence of Stx1 (0.05 pM) or 3AB (5 mM). To minimize the toxic effects on cells, in these experiments, the concentration of Stx1 was halved with respect to that used to completely inhibit DNA repair. As shown in the representative experiments in Figure 4A and 4B, mafosfamide strongly reduced the number of live Raji cells in a dose-dependent fashion, while Stx1 or 3AB, at the tested concentrations, did not elicit any effect on cell proliferation and viability. However, the number of viable cells was significantly lower in the presence of the combined treatment Stx1/mafosfamide (Figure 4A) to an extent comparable to that obtained with the reference DNA repair inhibitor 3AB (Figure 4B). It is worth noting that 3AB is a diffusible inhibitor that enters cells in a non-specific way, while Stx1, due to its receptor-mediated cell-type specific uptake, selectively hampers the DNA repair of cells harboring Gb3Cer/CD77 [6], such as the cancer Raji cells.

Conversely, it is well known that CD34+ human progenitor cells lacking the specific toxin receptor are spared [10]. The same occurred with HL-60 cells, which do not express Gb3Cer/CD77 and are sensitive to the toxic effect induced by mafosfamide, but not by Stx1. Indeed, the combined treatment did not potentiate the activity of mafosfamide on these cells (Figure 4C). In contrast, as shown in Figure 4D, the significantly different elevations (*p* < 0.05) of the straight lines obtained by plotting the mean percentage of live Raji cells from three independent experiments against the logarithm of drug concentrations after treatment with mafosfamide (*r* = −0.998) or mafosfamide/Stx1 (*r* = −0.982) confirm the Stx1-induced potentiating effect on mafosfamide acting on Raji cells. 

**Figure 5:**
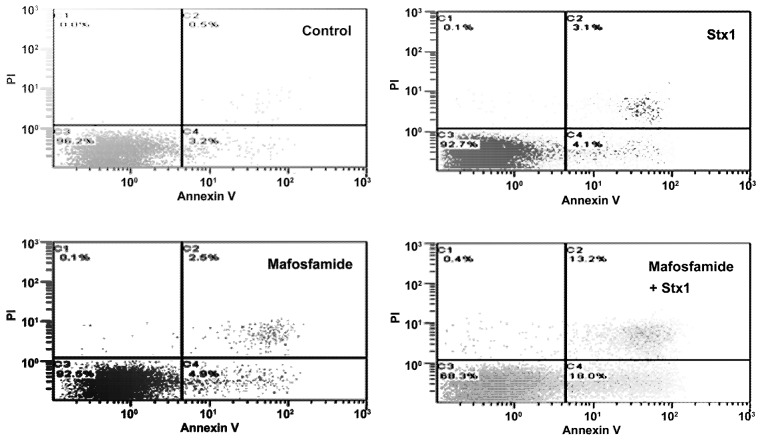
Determination of apoptosis in Raji cells after treatment with mafosfamide in combination with Stx1. Raji cells were treated as described in the upper panel of Figure 3. At the end of post-incubation, the cells were analyzed by flow cytometry after staining with annexin V and PI. Quadrant C1, necrosis; quadrant C2, late apoptotic cells; quadrant C3, live cells; and quadrant C4, early apoptotic cells. Mafosfamide and Stx1 were 1 μg/mL and 0.05 pM, respectively.

To investigate more closely the mechanism of cell death in treated Raji cells, double staining with propidium iodide (PI), as a probe for membrane damage and annexin V, as a probe for apoptosis, was performed after 48 h incubation. The annexin V assay is based on flow cytometric detection of FITC-conjugated annexin V bound to phosphatidylserine exposed on the outer leaflet of the plasma-membrane lipid bilayer of apoptotic cells. Annexin V-positive cells were classified as early apoptotic cells (Figure 5, quadrant C4), and annexin V- and PI-double-positive cells were considered to be late-apoptotic cells (Figure 5, quadrant C2). The results depicted in the representative cytograms in Figure 5 show that treatment of Raji cells with a low concentration of mafosfamide (1 μg/mL) induced only a modest significant increase (*p* < 0.005, *t*-test) in the number of early ((0.050 ± 0.001) × 10^6^) and late apoptotic cells ((0.026 ± 0.001) × 10^6^) with respect to controls ((0.031 ± 0.001) × 10^6^ and (0.006 ± 0.001) × 10^6^, respectively), confirming that the alkylating agent in this condition acted mainly on cell proliferation (Figure 4). The same occurred with Stx1 (0.05 pM), which slightly changed the number of early ((0.043 ± 0.003) × 10^6^; *p* < 0.05, *t*-test) and late apoptotic cells ((0.030 ± 0.001) × 10^6^; *p* < 0.005, *t*-test) with respect to controls (see above) when the toxin was administered alone (Figure 5). By contrast, the combined treatment of Raji cells with an inhibitor of DNA repair (Stx1) and an alkylating agent (mafosfamide) significantly (*p* < 0.005, two-way ANOVA) increased the number of early ((0.163 ± 0.024) × 10^6^) and late apoptotic cells ((0.120 ± 0.017) × 10^6^) with respect to single treatments (see above). There was also a corresponding reduction in live cells (quadrant C3) in mafosfamide/Stx1 treated cells. Necrosis (PI positive cells; quadrant C1) was almost absent under these conditions. Thus, the combined mafosfamide/Stx1 treatment triggers apoptosis in cancer cells.

To gain information on the reproductive integrity of Raji cells after the treatments outlined above, the clonogenic assay was performed. Cells scored as survivors were able to produce a colony of at least 50 cells within 10 days, indicating that they would give rise to viable offspring in an intact tissue. Raji cells were treated with mafosfamide (5–100 μg/mL) or with Stx1 (0.005–0.05 pM) under the experimental conditions shown in Figure 3. The clonogenic assay was then carried out by seeding 500 cells in 24-multiwell culture plates. The clonal efficiency of control Raji cells was 45.9% ± 6.5% (*n* = 4). In these conditions, both mafosfamide (Effective dose 50, ED50 = 11.5 ± 2.1 μg/mL; *r* = −0.979) and Stx1 (Effective concentration EC50 = 0.010 ± 0.001 pM, *r* = −0.995) assayed at different concentrations inhibited the formation of colonies. 

**Table 1:** Stx1 improves the inhibitory effect of mafosfamide on colony formation by Raji cells.

	Mafosfamide (μg/mL)		DRI ^a^	CI ^b^
	None	Stx1 (0.05 pM)		
**ED85 ^c^**	23	15	1.53	0.91
**ED90**	29	17	1.70	0.95
**ED98**	70	45	1.55	0.74

^a ^DRI, dose reduction index, calculated by Calculsyn version 2.1; ^b ^CI, combination index, calculated by Calculsyn version 2.1; ^c^ ED, effective dose, calculated by Calculsyn version 2.1.

Table 1 shows the ED85, ED90 and ED98 of mafosfamide in the absence and in the presence of 0.05 pM Stx1 (Calculsyn, version 2.1). The corresponding combination index (CI) and the dose reduction index (DRI) were calculated (Calculsyn, version 2.1). CI measures the degree of drug interaction in terms of additive effect (CI = 1) or synergistic effect (CI < 1). Under our experimental conditions, we consistently obtained CI values below unity indicating a synergistic cooperation between mafosfamide and Stx1. The results also showed that the dose of mafosfamide at any given effect was lower in the presence of the bacterial toxin (Table 1). 

## 3. Discussion

In the present study, we demonstrate for the first time that a quasi-non-toxic concentration of the bacterial toxin Stx1 potentiates the cytotoxic action of mafosfamide on Raji cells in a synergistic manner. Mafosfamide is a derivative of cyclophosphamide, which, unlike the latter, does not require metabolic activation to exert its antitumor activity [12]. Mafosfamide is widely used in the treatment of various hematological tumors and is also able to eliminate cancer cells before autologous bone marrow transplantation [18]. The DNA damaging activity of mafosfamide is recognized to play a prominent and causative role in its cytotoxic and anticancer activity [19]. Mafosfamide induces a variety of lesions to nuclear DNA, ranging from interstrand crosslinks, alkylative damages to DNA bases, DNA single and double strand breaks (SSBs and DSBs, respectively) [20]. Accordingly, we found that exposure of Raji cells or HL-60 cells to mafosfamide results in significant nuclear DNA damage. These lesions are scored by FHA as DNA SSBs, which are likely to reflect the majority of the lesions caused by the drug. Indeed, SSBs are directly detected, DSBs (due to the denaturing pH of FHA) are scored as SSBs, while base damages and interstrand crosslinks are elaborated and converted by repair enzymes into SSBs and then detected in this form by FHA [21]. Once produced, the DNA repair machinery of Raji cells or HL-60 cells is capable of removing most of mafosfamide lesions to DNA within a 4–6 h post-challenge incubation in drug-free medium. Interestingly, repair of the same lesions was greatly inhibited when Gb3Cer/CD77-expressing cells (Raji cells) had been pre-conditioned with sub-DNA damaging concentrations of Stx1, whereas the rate of DNA repair was unaffected in cells that do not harbor the specific toxin receptor (HL-60 cells). This finding is consistent with previous studies from our group showing that Stx1 inhibited the resealing of the DNA lesions caused by the oxidative agent H_2_O_2_ and by the alkylating agent methyl methane sulfonate in cultured sensitive cells, such as human endothelial cells [6]. We also found that the sensitivity of endothelial cells to methyl methane sulfonate was significantly increased by pre-treatment with Stx1 (unpublished observation). Indeed, inhibition of the repair of toxicologically relevant DNA lesions, such as those caused by antitumor agents, is known to potentiate their cytotoxicity and is considered as a valuable strategy to increase their therapeutic efficacy [22]. Interestingly, inhibition of DNA repair sensitizes various tumor cells to the cytotoxic activity of a wide number of alkylating agents, including either mafosfamide or its parental analogue cyclophosphamide [22,23,24,25]. Accordingly, preconditioning of Raji cells with a quasi-non-toxic dose of Stx1 significantly sensitized these cells to the action of mafosfamide, as assayed with the trypan blue exclusion assay. Using the more sensitive clonogenic assay, we found that Stx1 significantly decreased the ED85, ED90 and ED98 values of mafosfamide and; more interestingly, analysis of these data with Calculsyn software revealed that the interaction between the two agents was synergistic. This notion is further supported by the observation that, while mafosfamide at the lowest concentration tested (1 μg/mL) is basically cytostatic to Raji cells (Figure 4), it becomes cytotoxic toward Stx-primed cells, inducing significant apoptotic cell death. It is worth noting that the concentrations of Stx1 (0.05–0.1 pM) used throughout these experiments were very low (45–90 toxin molecules/cell). At such doses, it is not cytotoxic and only slightly inhibitory to protein synthesis. In other words, Stx1 synergistically interacts with mafosfamide at concentrations lower than those previously selected for *ex vivo* bone marrow purging [26], which, unfortunately, are regarded as unsafe for clinical purposes. 

The synergistic cooperation between the two agents is likely to depend on the Stx1-mediated inhibition of the DNA repair efficiency of mafosfamide-injured cells. Indeed, we also show that in the presence of 3AB, selected as a reference DNA repair inhibitor acting on PARP, the rejoining of mafosfamide-induced DNA lesions in Raji cells was impaired to an extent similar to that caused by Stx1. This response was also associated with an increase in the sensitivity of Raji cells to mafosfamide toxicity. Thus, it is likely that the persistence of unrepaired mafosfamide-induced DNA breaks is causally linked to the increased susceptibility of Raji cells to the alkylating agent. Although the precise mechanism(s) whereby Stx1 impairs DNA repair machinery was not extensively investigated and is beyond the scope of the present study, we hypothesized that it might involve the inhibition of PARP-dependent processes [6]. Indeed, it has been shown that ricin, the plant toxin homologous to Stx1, catalyzes the release of adenines from poly(ADP-ribosyl)ated PARP *in vitro* [27]; it is worth noting that an extensive depurination might promote precocious degradation of poly(ADP-ribose) polymers [27]. Since the effects of PARP directly depend on the extent of poly(ADP-ribosyl)ation, a post-translational modification of proteins involving synthesis of ADP-ribose polymers on target proteins, it is reasonable to hypothesize that ricin and related toxins might accelerate *in vivo* deadenylation of either automodified PARP or of specific acceptor poly(ADP-ribosyl)ated proteins. This would, in turn, disrupt PARP-dependent nuclear processes, including DNA repair. The hypothesis that Stx1 may act via a PARP-dependent mechanism is indirectly supported by the following observations: (i) 3AB promotes effects quantitatively similar to those caused by Stx1 and (ii) the cooperative action of DNA repair enzymes involved in alkylative damage repair depends on the extent of poly(ADP-ribosyl)ation [6,28].

Purging of cancer cells from bone marrow before transplantation is a widely used and effective strategy in the treatment of lymphomas and leukemias [18]. Stx1 targets cells that express the glycolipid Gb3Cer/CD77, whose frequent occurrence on tumor cells derived from patients with hematological cancers (follicular lymphoma, multiple myeloma, chronic lymphocytic leukemia), along with its absence on human CD34+ hematopoietic stem cells, justify the *ex vivo* use of cytotoxic concentrations of Stx1 in purging CD77+ tumor cells from autologous stem cell transplants [10,29]. However, the fact that Gb3Cer/CD77 is expressed also by many other cell types in the body, combined with the very high intrinsic toxicity of Stx1 [1,13,14], discourages the use of the toxin even in such an *ex vivo* setting, unless quasi-non-toxic concentrations of Stx1 are used in combination with an anticancer drug, as suggested in the present paper.

## 4. Experimental Section

### 4.1. Drugs

The Stx1-producer *E. coli* C600 (H19J) was kindly supplied by Dr Alison O’Brien (Department of Microbiology and Immunology, Uniformed Services University of the Health Sciences, Bethesda, MD, USA). Stx1 was purified by receptor analogue affinity chromatography [30] on globotriose—Fractogel (IsoSep AB, Lund, Sweden). The purity of the toxin was verified by SDS-polyacrylamide gel electrophoresis. Toxicity was assayed by measuring the inhibition of translation (IC_50_ = 0.9 pM) in human umbilical vein endothelial cells treated with the toxin, as previously described [4]. Mafosfamide was kindly provided by Prof. Vittorio Rizzoli (Sezione di Ematologia e Centro Trapianti Midollo Osseo, Università di Parma, Parma, Italy).

### 4.2. Culture of Cells

Raji cells, a cell line presenting genomic stability [31] even though derived more than 45 years ago from a patient with Burkitt lymphoma [32], were kindly provided by Prof. Andrea Bolognesi (Dipartimento di Patologia Sperimentale, Università di Bologna, Bologna, Italy). HL-60 cells, a generous gift of Prof. Carmela Fimognari (Dipartimento di Farmacia, Università di Bologna, Bologna, Italy), were purchased by Istituto Zooprofilattico (Brescia, Italy). Cells were maintained in RPMI 1640 medium (Lonza, Walkersville, MD, USA) containing antibiotics (60 U/mL penicillin, 60 μg/mL streptomycin, Cambrex, Walkersville, MD, USA) and supplemented with 4 mM L-glutamine (Sigma Aldrich, St. Louis, MO, USA) and 10% or 20% fetal bovine serum (Lonza, Walkersville, MD, USA), with Raji cells or HL-60 cells, respectively. In the following experiments, Raji cells or HL-60 cells (1 × 10^6^) were seeded in 1.5 mL complete growth medium in 3.5 cm diameter dishes, incubated overnight and then treated, as described in Figures and their legends. Cultures were kept in an incubator at 37 °C in a water-saturated 5% CO_2_ atmosphere in air.

### 4.3. Protein Synthesis Measurement in Whole Cells

Cells were treated, as indicated in the legends to Figure 1 and Figure 2 and in the panel of Figure 2. At the end of the incubation period, protein synthesis was measured as the rate of incorporation of radiolabeled leucine during a 30 min incubation of the cells at 37 °C in 1.5 mL of complete medium containing 2 μCi of [^3^H]leucine (62 Ci/mmol; Amersham Biosciences, Bucks, UK). The cells were centrifuged 5 min at 200 × *g* and washed three times with 0.5 mL of ice-cold phosphate buffered saline (PBS) containing 10 mM leucine. The cellular pellets were treated with 0.5 mL of ice-cold 10% (*v*/*v*) trichloroacetic acid, followed by 5 min of incubation on ice and centrifugation for 10 min at 13,000 × *g*. The procedure was repeated three times. Finally, the precipitated cellular proteins were resuspended in 200 μL of 0.2 M KOH, and the radioactivity was measured in a liquid scintillation counter. In each experiment, blank values obtained by incubating the cells in the presence of radiolabeled leucine (see above) for 30 min at 0 °C were subtracted. 

### 4.4. Fast Halo Assay

DNA damage and repair were analyzed with the FHA, conducted as previously described [17,21]. Briefly, after the treatments, the cells were resuspended at 4.0 × 10^4^/mL in ice-cold PBS containing 5 mM EDTA; 25 μL of this cell suspension were diluted with an equal volume of 2% low melting agarose in PBS and immediately sandwiched between an agarose-coated slide and a coverslip. The latter were removed, and the slides were immersed in 300 mM NaOH for 15 min at room temperature. Ethidium bromide (10 μg/mL) was directly added to NaOH during the last 5 min of incubation. The ethidium bromide-labeled DNA was visualized using a Leica DMLB/DFC300F fluorescence microscope (Leica Microsystems, Wetzlar, Germany) equipped with an Olympus Colorview IIIU CCD camera (Olympus Italia Srl, Segrate, Italy), and the resulting images were digitally recorded on a PC and processed with image analysis software (Scion Image, Scion Corporation, Frederick, MD, USA). The amount of fragmented DNA diffusing out of the nuclear cage, *i.e.*, the extent of strand scission, was quantified by calculating the nuclear spreading factor, which represents the ratio between the total area (halo and nucleus) and that of the nucleus. Data are expressed as RNSF, calculated by subtracting the nuclear diffusion factor of control cells from those of treated cells. 

### 4.5. Determination of Cell Survival and Apoptosis

Cell proliferation and viability were determined by counting the cells after proper dilution; cell death was monitored by counting cells permeated by 0.06% trypan blue. Apoptosis was evaluated by assessing the annexin V binding to phosphatidylserine exposed on the outer leaflet of the plasma membrane of apoptotic cells and by evaluating, simultaneously, the exclusion of PI (ApoScreen Annexin V apoptosis kit-FITC; Beckman Coulter, Miami, FL, USA) by flow cytometry.

### 4.6. Clonogenic Assay

The effect of exposure of Raji cells to Stx1 and mafosfamide, either alone or in combination, was evaluated by a clonogenic assay. Cells were incubated with Stx1 and mafosfamide, according to the experimental set described in Figure 3 (left upper panel). After the washing procedures, 500 colony-forming Raji cells were cultured in 500 μL Iscove’s modified Dulbecco’s medium containing 1.4% methylcellulose, 25% fetal bovine serum, 2% bovine serum albumin, 2 mM L-glutamine and 50 μM 2-mercaptoethanol (Human methylcellulose base media; R&D Systems Inc., Minneapolis, MN, USA). Cultures were incubated at 37 °C in humidified atmosphere containing 5% CO_2_ for 10 days. Colonies of more than 50 cells were scored visually under a microscope by three independent observers. Each experimental point was performed in duplicate and the experiments were repeated four times. 

### 4.7. Statistics

Differences in continuous variables were compared with *t-*tests or with two-way ANOVA after verifying the normality of their distribution. A value of *p* < 0.05 was considered statistically significant. Correlation between variables was assessed by the Pearson correlation coefficient. Differences in slopes and elevations of different straight lines were tested by linear regression.

## 5. Conclusions

A strategy based on the combination of a DNA repair inhibitor and an anticancer agent has been exploited to ameliorate the clinical outcomes of antitumor therapies [22]. In particular, PARP inhibitors have gained recent attention, since they augment head and neck cancer cell susceptibility to cetuximab *in vitro* [33]. This therapeutic agent targets the epidermal growth factor receptor; thus, the combined treatment seems feasible in different human tumors with aberrant epidermal growth factor receptor signaling. In this respect, our findings pose a rationale for the evaluation and possible utilization of an approach involving association between mafosfamide and Stx1, in a specific, but clinically relevant, situation; namely, selective purging of Gb3Cer/CD77+ tumor cells before autologous bone marrow transplantation.
